# Isolation, culture and phenotypic characterization of human sweat gland epithelial cells

**DOI:** 10.3892/ijmm.2014.1851

**Published:** 2014-07-14

**Authors:** YUNHE GAO, MEIYING LI, XUEYAN ZHANG, TINGTING BAI, GUANFAN CHI, JIN YU LIU, YULIN LI

**Affiliations:** The Key Laboratory of Pathobiology, Ministry of Education, College of Basic Medical Sciences, Jilin University, Changchun, Jilin 130021, P.R. China

**Keywords:** sweat glands, epithelial cells, culture medium, serum-free, stem cells, leucine-rich repeat-containing G protein-coupled receptor 5

## Abstract

Sweat gland epithelial cells (SGECs) have been identified as essential for the regeneration of sweat glands and for the construction of skin substitutes containing skin appendages. Consequently, the isolation, culture and phenotypic characterization of SGECs are of paramount importance. In the present study study, human sweat glands were isolated by pipetting under a phase contrast microscope following digestion with collagenase type I. Subsequently, a microscopic organ culture technique was used for the primary culture of human SGECs, and the culture conditions were modified in order to achieve optimal cell growth status. Primary SGECs were identified based on their expression of markers specific for sweat glands, including carcinoembryonic antigen (CEA), CK7, CK8, CK14, CK15, CK18 and CK19. We explored the possible presence of stem cells in human sweat glands by detecting their expression of leucine-rich repeat-containing G protein-coupled receptor 5 (LGR5). Primary SGECs achieved a good growth state when cultured under serum-free conditions. After one passage, the cells cultured in keratinocyte serum-free medium with 1% fetal bovine serum (FBS) still showed a prominent proliferative activity. Phenotypic analysis by immunofluorescence microscopy, reverse transcription-polymerase chain reaction (RT-PCR), and western blot analysis demonstrated the expression of sweat gland-specific markers, including CEA, CK7, CK8, CK14, CK15, CK18 and CK19. In addition, RT-PCR and immunochemistry detected the expression of LGR5. In comparison with traditional serum-containing conditions, serum-free culture provides the preferred culture conditions for human SGECs. LGR5 is a novel marker that identifies human sweat gland-derived stem cells.

## Introduction

Engineered skin substitutes play a potentially important role in the treatment of extensive burns and chronic wounds (1,2). However, existing skin substitutes have not completely replicated native human skin, since they lack skin appendages (1). Sweat glands, one of the skin appendages, serve a number of vital functions, including the maintenance of homeostasis and thermal regulation (3). Therefore, the regeneration of sweat glands has become a recent research goal in skin tissue engineering.

Approaches to sweat gland regeneration include: i) studies on the tubular morphogenesis *in vitro* of human eccrine sweat gland epithelial cells (hESGECs) cultured in Matrigel (4); ii) regeneration of functional sweat gland-like structures following the implantation of bone mesenchymal stem cells co-cultured with sweat gland cells (5); iii) constitution of engineered skin constructs with sweat glands by incorporating sweat gland cell-microsphere complexes *in vitro* (6). As a consequence of this research effort, sweat gland epithelial cells (SGECs) have been identified as essential for the reconstitution of sweat glands *in vitro*, as well as for their regeneration *in situ*. The aim of the present study was to improve the methods for the isolation and culture of hSGECs, in order to explore the possible presence of sweat gland-derived stem cells and thereby provide an experimental basis for the reconstruction of sweat glands and the creation of appendage-containing engineered skin models.

In a previous study, a serum-containing medium was used to culture hSGECs (5). However, when we repeated these experiments, we found that there were technical difficulties with this method, including the keratinization of cultured cells and fibroblast contamination.

To address these issues, we used enzymatic digestion methods and serum-free medium to maintain the undifferentiated state and harvest optimal numbers of hSGE (stem) cells. SGE (stem) cells were verified by the identification of markers specific for sweat glands. In the present study, we present a systematic isolation procedure and modified culture conditions for the isolation and growth of hSGE (stem) cells.

## Materials and methods

### Isolation of human sweat glands

Full thickness skin samples were obtained from the abdomen and the upper arm area of surgical patients who were between 15 to 43 years of age. Following the removal of subcutaneous fat under aseptic conditions, the skin was rinsed with phosphate-buffered saline (PBS) containing 100 μ/ml penicillin and 100 μg/ml streptomycin (HyClone Laboratories, Inc., Logan, UT, USA). A part of the sample was fixed in 10% formalin for the preparation of paraffin sections and the remainder was minced into 1-mm^3^ sections and digested overnight with 0.1% collagenase type I (Gibco, Grand Island, NY, USA) in a 37°C/5% CO_2_ incubator. On the following day, the sweat glands were isolated with a pipettor under a clean ultraviolet-sterilized phase contrast microscope.

### Primary culture of intact glands

Firstly, the glands were transferred to DMEM/F-12 (1:1) medium (Gibco) containing 5% fetal bovine serum (FBS; HyClone). They were then cultured in sweat gland culture medium consisting of DMEM/F-12 (1:1) medium supplemented with 5% (v/v) FBS, 100 μ/ml penicillin and 100 μg/ml streptomycin, 10 ng/ml endothelial growth factor (EGF; Promega Corp., Madison, WI, USA), 2 mM L-glutamine (Sigma, St. Louis, MO, USA), 1 ml/100 ml insulin-transferring-sodium (Sigma), 2 nM/ml triiodothyronine (Sigma) and 0.4 g/ml hydrocortisone 21-hemisuccinate (Sigma).

### Primary serum-containing culture and purification

The primary SGECs were cultured in sweat gland culture medium and the purification was performed as follows: the medium was removed, the cells were rinsed with PBS twice, then digested with 0.25% trypsin (Invitrogen Life Technologies, Carlsbad, CA, USA) and 0.02% EDTA (Tong Zheng, Beijing, China). When most of the fibroblasts had retracted and the SGECs remained adherent, an equal volume of culture medium containing 10% (v/v) FBS was added to terminate the digestion. Fibroblasts were detached with a pipettor and removed by washing with PBS. Fresh sweat gland culture medium was added for further culture.

### Primary serum-free culture and cell passaging

When the sweat gland tissues had adhered to the bottom of the culture well and a few cells had grown out from the explants, serum-free keratinocyte medium containing 50 μg/ml bovine pituitary extract (BPE) and 5 ng/ml EGF (all from Invitrogen Life Technologies) were added to replace the sweat gland culture medium. The SGECs were then cultured in a 37°C/5% CO_2_ incubator. The medium was changed every 2–3 days.

The primary SGECs were passaged when they reached approximately 60–80% confluence. The cells were rinsed twice with PBS, and were then digested with 0.25% trypsin and 0.02% EDTA in a 37°C/5% CO_2_ incubator for 5–8 min. An equal volume of culture medium containing 10% (v/v) FBS was added to terminate the digestion. The liquid was transferred into a centrifuge tube, centrifuged at 1,000 rpm/min for 5 min, and the cell pellets were collected and resuspended in serum-free keratinocyte medium containing 50 μg/ml BPE, 5 ng/ml EGF and 1% FBS.

### Immunofluorescent histochemical staining

After dewaxing and hybration, the sectioned samples were blocked with 10% FBS. The sections were incubated with primary antibodies at 4°C overnight. The antibodies used were anti-CD7, anti-CD8, anti-CD14, anti-CD15, anti-CD18, anti-CD19 and anti-carcinoembryonic antigen (CEA) (Abcam, Cambridge, MA, USA). The sections were then incubated with Alexa Fluor 488-conjujated anti-mouse/anti-rabbit secondary antibodies (Cell Signaling Technology, Inc., Danvers, MA, USA), for 1 h at room temperature. Nuclei were stained with Hoechst 33342 (Invitrogen Life Technologies).

### Immunohistochemical staining

After dewaxing and hydration, the sectioned samples were treated with 3% H_2_O_2_ for 10 min to block endogenous peroxidase activity. Subsequently, 10% normal goat serum was used to block non-specific binding. The sections were incubated with primary antibody against leucine-rich repeat-containing G protein-coupled receptor 5 (LGR5) at 4°C overnight, followed by incubation with goat anti-mouse/rabbit secondary antibodies for 10 min at room temperature. The Ultra-Sensitive™ S-P detection system kit (Maixin, Fuzhou, China) was used with 3-amino-9-ethylarbazole (AEC) (Boster Biological Technology, Ltd., Wuhan, China) as the chromogenic substrate for visualization. Nuclei were counterstained with hematoxylin.

### Immunofluorescent cytochemical staining

The SGECs on coverslips were fixed in 4% paraformaldehyde for 10 min, permeabilized with 0.1% Triton X-100 (Sigma) for 20 min at room temperature. Subsequently, 10% FBS was used to block non-specific binding. The SGECs were then incubated with primary antibodies at 4°C overnight. The antibodies that were used were the following: anti-CD7, anti-CD8, anti-CD14, anti-CD15, anti-CD18, anti-CD19, anti-CEA and anti-LGR5 (Abcam). The cells were then incubated with Alexa Fluor 488-conjujated anti-mouse/anti-rabbit secondary antibodies (Cell Signaling Technology, Inc.) for 1 h at room temperature. Nuclei were stained with Hoechst 33342.

### Reverse transcription-polymerase chain reaction (RT-PCR)

Total RNA from the cells was isolated using TRIzol (Invitrogen Life Technologies) reagent according to the manufacturer’s instructions. cDNA was synthesized from 500 ng total RNA using the Takara RT-PCR AMV 3.0 kit. PCR was carried out with 1 μl cDNA in a 20 μl reaction volume using a PCR kit (Kangweishiji Biotech Co., Ltd., Beijing, China). A negative control was established by using H_2_O as the template. PCR products were detected by 1.5% agarose gel electrophoresis. The primers used were as follows: CK7 (forward, 5′-GCATCAT CGCTCAGGTCAA-3′ and reverse, 5′-TCACGGCTCCCA CTCCAT-3′); CK8 (forward, 5′-TGACCGACGAGATAAA CTTCC-3′ and reverse, 5′-CTTGGCGTTGGCATCCTTA-3′); CK14 (forward, 5′-TGAGCCGCATTCTGAACGAG-3′ and reverse, 5′-GATGACTGCGATCCAGAGGA-3′); CK15 (forward, 5′-TCTGCTAGGTTTGTCTCTTCAGG-3′ and reverse, 5′-CCA GGGCACGTACCTTGTC-3′); CK18 (forward, 5′-TGGTCACC ACACAGTCTGCT-3′ and reverse, 5′-CCAAGGCATCACCAA GATTA-3′); CK19 (forward, 5′-AGGTGGATTCCGCTCCG GGCA-3′ and reverse, 5′-ATCTTCCTGTCCCTCGAGCA-3′); CEA (forward, 5′-GACGCAAGAGCCTATGTATG-3′ and reverse, 5′-GGCATAGGTCCCGTTATTA-3′); LGR5 (forward, 5′-CTCTTCCTCAAACCGTCTGC-3′ and reverse, 5′-CACT CCAAATGCACAGCACT-3′); and GAPDH (forward, 5′-TGT TGCCATCAATGACCCCTT-3′ and reverse, 5′-CTCCACGA CGTACTCAGCG-3′).

### Western blot analysis

Proteins (20 μg/lane) were fractionated by SDS-PAGE and electrotransferred onto polyvinylidene difluoride membranes. The blots were first incubated for 1 h in a blocking buffer consisting of 0.1% Tween-20 (Invitrogen Life Technologies) and 5% non-fat powdered milk, then incubated with a primary antibody at 4°C overnight. The antibodies that were used were as follows: anti-CD7, anti-CD8, anti-CD14, anti-CD15, anti-CD18, anti-CD19 (Abcam) and anti-β-actin (Cell Signaling Technology, Inc.). A horseradish peroxidase-conjugated, goat anti-mouse/anti-rabbit secondary antibody (Zhongshan Jinqiao Biotechnology Co., Ltd., Beijing, China) then was used and antigen-antibody complexes were detected by chemiluminescence using the BeyoECL Plus kit (BiYunTian Biotechnology Research Laboratory, Haimen, China).

## Results

### Isolation of human sweat glands and cultivation of SGECs

**Figure 1:**
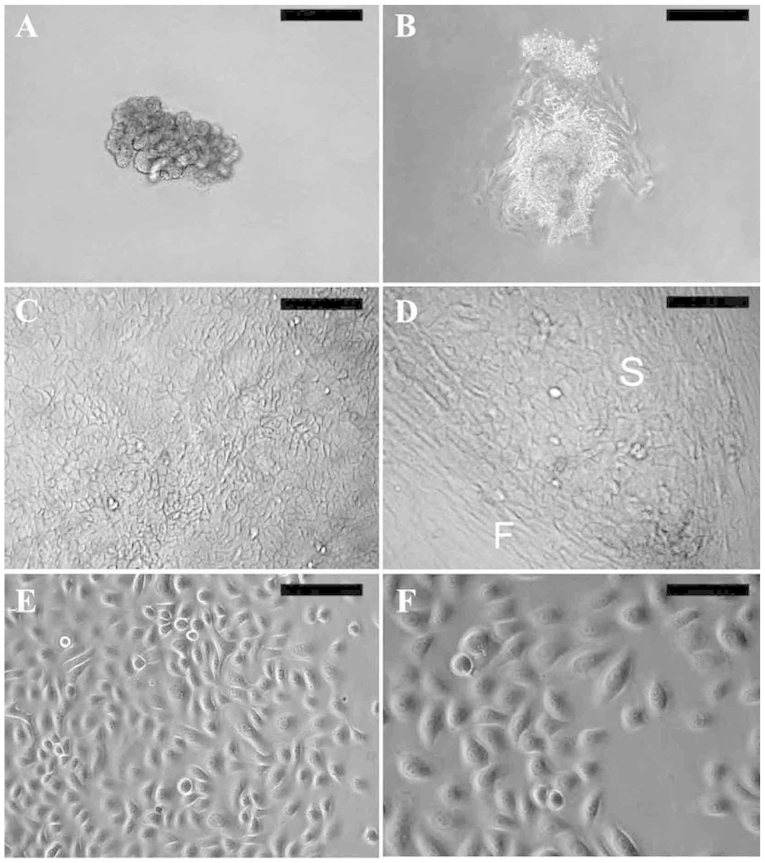
Isolation and cultivation of human sweat gland epithelial cells (hSGECs). (A) Isolation of human sweat glands following collagenase digestion. (B) Typical morphology of epithelial cells growing out from a human sweat gland fragment. Morphological characteristics of primary SGECs cultured under serum-containing conditions. (C) SGECs resembled keratinized cells and (D) the SGECs (S) usually contaminated by fibroblasts (F). (E) Morphological characteristics of primary SGECs cultured under serum-free conditions. (F) Morphological characteristics of first-passage SGECs cultured in keratinocyte serum-free medium with 1% fetal bovine serum (FBS). SGECs demonstrated good growth and displayed an epithelial-like morphology with a rounded large nucleus. Scale bar: (A) 200 μm, (B–E) 100 μm, (F) 50 μm.

Following overnight digestion with 0.1% collagenase type I, the sweat glands were dissociated from adjacent connective tissue. Preparations of simple branched tubular glands mainly consisted of the secretory portion (Fig. 1A). Following the culture of the whole glands in sweat gland culture medium for 2–5 days, typical epithelial cells grew out from the explants, assuming a cobblestone morphology (Fig. 1B). After continued growth in sweat gland culture medium, the primary SGECs resembled keratinized cells (Fig. 1C) and were usually contaminated by fibroblasts (Fig. 1D).

The primary SGECs assumed a good growth state after replacing the sweat gland culture medium with keratinocyte serum-free medium. They displayed an epithelial-like morphology with a rounded cell shape and a large nucleus (Fig. 1E), and grew rapidly; a confluent monolayer was formed approximately 1 week later. Few fibroblasts survived under serum-free conditions. After one passage, cell propagation was performed in keratinocyte serum-free medium with 1% FBS, and the cells still showed prominent proliferative activity (Fig. 1F).

### Immunofluorescent histochemical staining

**Figure 2:**
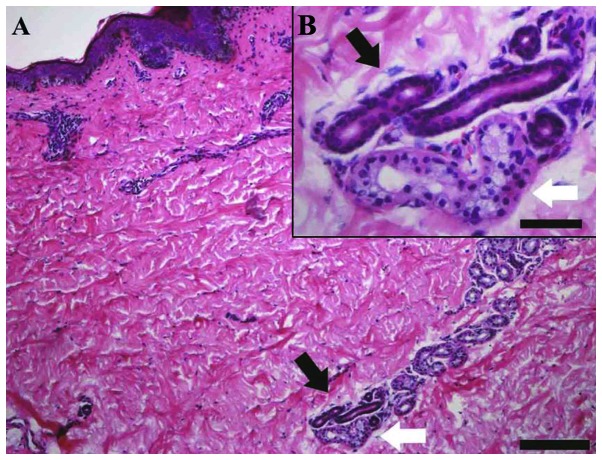
Hematoxylin and eosin (H&E) staining of adult skin paraffin sections revealed the anatomic characteristics of eccrine sweat glands. (A) Sweat glands containing the secretory portion (white arrows) and the duct (black arrows). Scale bar, 200 μm. (B) The insert shows high magnification. Scale bar, 50 μm.

**Figure 3:**
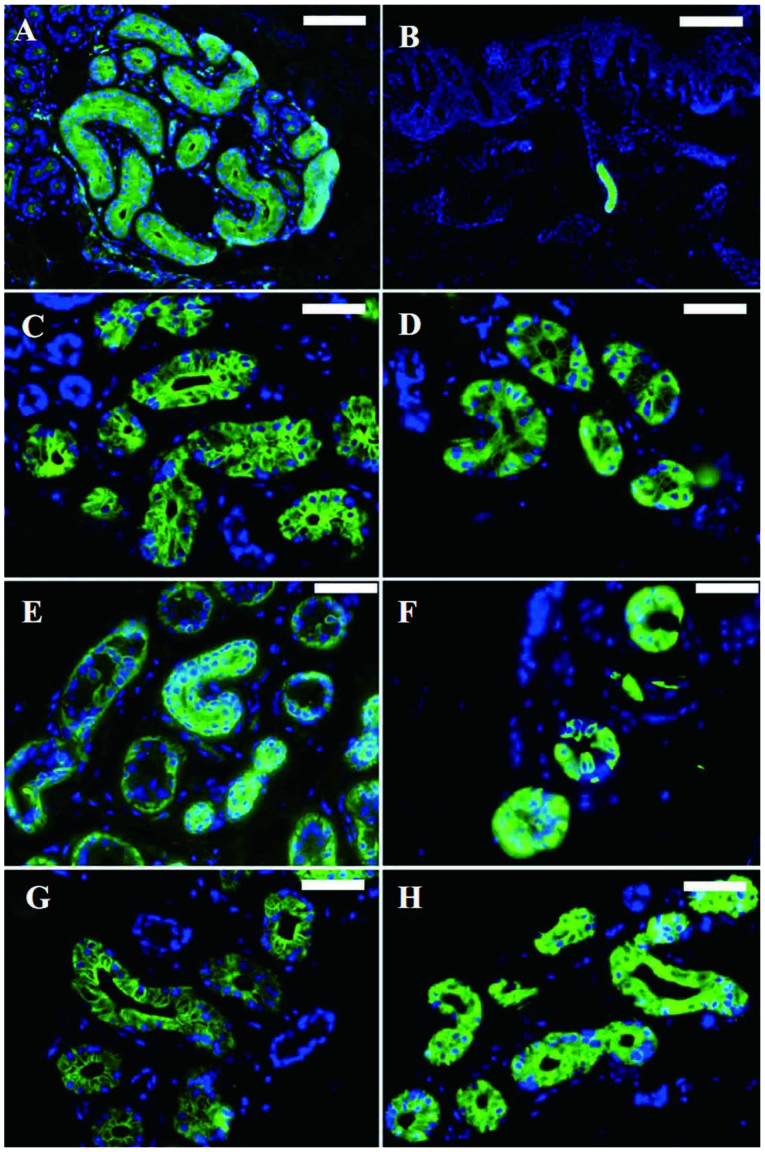
Immunofluorescent staining revealed the expression of markers specific for sweat glands in the human sweat gland epithelial cells (hSGECs). Carcinoembryonic antigen (CEA) was expressed in (A) the secretory portion and (B) the ductal portion. (C) CK7, (D) CK8, (F) CK15, (G) CK18 and (H) CK19 were expressed in the secretory portion. (E) CK14 was expressed in both the secretory portion and ductal portion. Nuclei were stained with Hoechst 33342. Scale bar: (A) 100 μm, (B) 200 μm, (C–H) 50 μm.

Hematoxylin and eosin (H&E) staining of adult skin paraffin sections revealed the anatomic characteristics of eccrine sweat glands. The secretory portion was located in the dermis and subcutaneous tissue, with a distinct lumen (Fig. 2, white arrows). The duct consists of 2 layers of cuboidal cells (Fig. 2, black arrows). Immunofluorescent histochemical staining revealed that CEA (Fig. 3A and B) and CK14 (Fig. 3E) were expressed in both the secretory and the ductal portion, and that CK7 (Fig. 3C), CK8 (Fig. 3D), CK15 (Fig. 3F), CK18 (Fig. 3G) and CK19 (Fig. 3H) were expressed in the secretory portion.

### Immunofluorescent staining of SGECs

**Figure 4:**
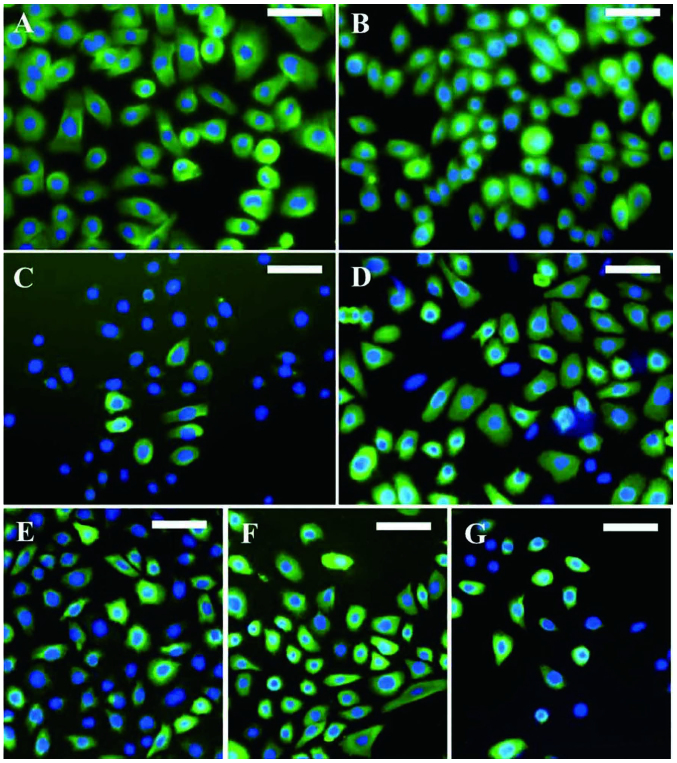
Immunofluorescent staining revealed the expression of markers specific for sweat glands in the cultured human sweat gland epithelial cells (hSGECs). Staining revealed the positive expression of (A) carcinoembryonic antigen (CEA), (B) CK7, (C) CK8, (D) CK14, (E) CK15, (F) CK18 and (G) CK19. Nuclei were stained with Hoechst 33342. Scale bars, 50 μm.

Immunofluorescent staining revealed that the SGECs were positive for CEA (Fig. 4A), CK7 (Fig. 4B), CK8 (Fig. 4C), CK14 (Fig. 4D), CK15 (Fig. 4E), CK18 (Fig. 4F) and CK19 (Fig. 4G), in accordance with the skin sections.

### RT-PCR and western blot analysis

**Figure 5:**
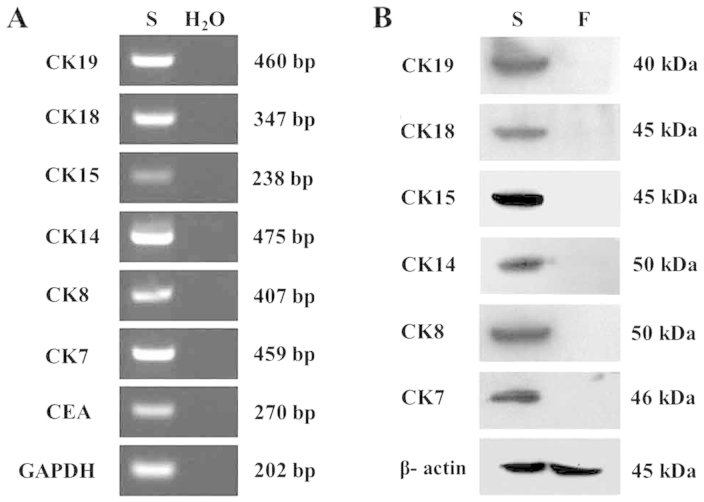
Identification of of markers specific for sweat glands in human sweat gland epithelial cells (hSGECs) by reverse transcription-polymerase chain reaction (RT-PCR) and western blot analysis. (A) Carcinoembryonic antigen (CEA), CK7, CK8, CK14, CK15, CK18 and CK19 were detected in SGECs (S) at the mRNA level; a negative control was established by using H_2_O as the template. (B) CK7, CK8, CKl4, CKl5, CKl8, CKl9 were detected in the SGECs (S) by western blot analysis, compared with the fibroblasts (F) in which these markers were not found.

The markers specific for sweat glands were identified by RT-PCR. CEA, CK7, CK8, CK14, CK15, CK18 and CK19 were detected at the mRNA level (Fig. 5A). Western blot analysis with keratin subunit-specific monoclonal antibodies confirmed the expression of CK7, CK8, CK14, CK15, CKl8 and CKl9 in the SGECs. The expression of these specific markers distinguished these cells from fibroblasts (Fig. 5B).

### Expression of LGR5 in SGECs

**Figure 6:**
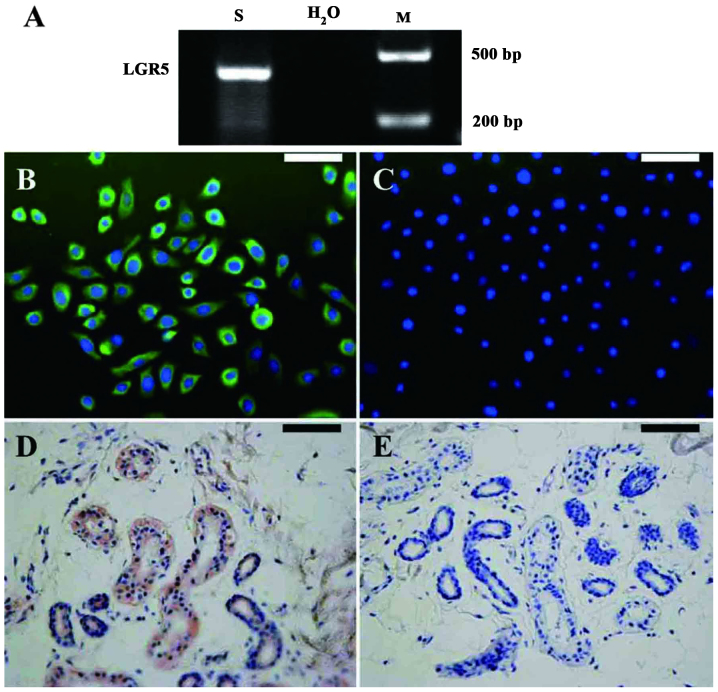
Reverse transcription-polymerase chain reaction (RT-PCR) and immunohistochemical staining revealed the expression of leucine-rich repeat-containing G protein-coupled receptor 5 (LGR5) in human sweat gland epithelial cells (hSGECs). (A) The specific expression of LGR5 was confirmed in the SGECs by RT-PCR; a negative control was established by using H_2_O as the template. (B) Immunofluorescent staining revealed the specific expression of LGR5 in human SGECs. (C) Negative control. (D) The epithelial cells in sweat glands of the skin tissue also expressed LGR5. (E) Negative control. Scale bars: (B and C) 50 μm, (D and E) 100 μm.

We found that the SGECs expressed LGR5, as shown by RT-PCR (Fig. 6A) and immunocytochemistry (Fig. 6B and C), known as the stem cell marker of intestinal cells. To confirm our results, we stained the sweat gland tissue in the skin. Consistent with the immunocytochemistry results, the epithelial cells in the sweat glands of the skin tissue also expressed LGR5 (Fig. 6D and E).

## Discussion

hSGECs are essential for the reconstitution of sweat glands *in vitro* (4), as well as for their regeneration *in situ* (5). Moreover, sweat gland cells can reconstitute a functional, stratified epidermis (7) and participate in the construction of engineered skin constructs with sweat glands (6). Therefore, an optimized method for isolating and culturing hSGE (stem) cells is important for the reconstruction of sweat glands and the creation of skin appendage-containing engineered skin models.

In the present study, intact sweat glands were isolated from small sections of skin by pipetting under a phase contrast microscope following overnight digestion with 0.1% collagenase type I. Subsequently, the intact glands were cultured, making it possible to determine the source of cells for primary culture. Initially, we tried a traditional serum-containing culture medium (5); however, the primary hSGECs resembled keratinized cells and were usually contaminated by fibroblasts. Consequently, we established a serum-free culture method and obtained primary hSGECs with a good growth state. After one passage, the cells cultured in keratinocyte serum-free medium with 1% FBS still showed proliferative activity. The SGECs formed a ‘dome-like’ structure after growing into a confluent monolayer that corresponded with the biological characteristics of glandular epithelial cells which may paly a role in ion transmembrane transportation (8,9). Immunofluorescence microscopy, RT-PCR and western blot analysis demonstrated the expression of specific phenotypic surface markers, including CEA, CK7, CK8, CK14, CK15, CK18, and CK19 in cultured SGECs. We concluded that serum-free culture provides an optimal culture condition for hSGECs.

Sweat glands are detected in embryos at 14–16 weeks and they reach maturation in 24–week embryos (10). It is generally accepted that there is no sweat gland formation after birth. The markers expressed in human sweat glands are also different at each developmental stage of embryogenesis. In normal adult skin tissue, CK7, CK8, CK14, CK15, CK18 and CK19 are expressed in the secretory portion (5,11).

CEA is an important marker for the diagnosis of colorectal cancer and other types of cancer, bu also shows limited tissue expression in normal adult tissues, including columnar epithelial cells and goblet cells in the colon, mucous neck cells and pyloric mucous cells in the stomach, secretory epithelia and duct cells of sweat glands (12). That means CEA is only expressed in sweat glands in normal adult skin tissue and is therefore considered as a relatively specific marker for the identification of sweat gland cells.

Thus, the high expression of CEA, CK7, CK8, CK14, CK15, CK18 and CK19 may be considered as an index for identifying adult SGECs (5,11–12). According to cell morphology and the expression of tissue-specific markers, we came to the conclusion that SGECs were successfully isolated and passaged.

It has been proven that there are multipotent stem cells in the hair follicle bulge areas and that they contribute to epidermal regeneration following skin injuries (13). Human glabrous skin, which completely lacks hair follicles but contains abundant sweat glands, such as the skin area of the palms, also has epidermal regenerative potential (7), suggesting the possible presence of stem cells in human sweat glands.

Sweat glands contain 4 segments: an intraepidermal duct, an intradermal ‘straight’ duct, an intraglandular ‘coiled’ duct and a secretory portion (14). Label-retaining cells are located in the intraglandular ‘coiled’ duct and secretory segments. These cells express CK15, a positive marker for stem cells residing in the hair follicle bulge areas (15,16). In this study, the cultured SGECs were mainly derived from the secretory portion and we found clear evidence that they strongly expressed CK15. To the best of our knowledge, for the first time, the expression of LGR5 was detected by RT-PCR and immunohistochemistry in human sweat glands. This is a novel marker that specifically identifies intestinal epithelial stem cells and hair follicle stem cells (17,18). The expression of CK15 and LGR5 indicates that CK15 and LGR5 may also represent markers of human sweat gland-derived stem cells.

In conclusion, SGE (stem) cells were successfully isolated by collagenase digestion and harvested in culture in serum-free culture medium. The present study provides an experimental basis for the reconstruction of sweat glands and the creation of skin appendage-containing engineered skin models.
